# The Effect of SGLT2 Inhibition on Brain-related Phenotypes and Aging: A Drug Target Mendelian Randomization Study

**DOI:** 10.1210/clinem/dgae635

**Published:** 2024-09-13

**Authors:** Zhihe Chen, Xueyan Wu, Qianqian Yang, Huiling Zhao, Hui Ying, Haoyu Liu, Chaoyue Wang, Ruizhi Zheng, Hong Lin, Shuangyuan Wang, Mian Li, Tiange Wang, Zhiyun Zhao, Min Xu, Yuhong Chen, Yu Xu, Jieli Lu, Guang Ning, Weiqing Wang, Shan Luo, Shiu Lun Au Yeung, Yufang Bi, Jie Zheng

**Affiliations:** Department of Endocrine and Metabolic Diseases, Shanghai Institute of Endocrine and Metabolic Diseases, Ruijin Hospital, Shanghai Jiao Tong University School of Medicine, Shanghai 200025, China; Shanghai National Clinical Research Center for Metabolic Diseases, Key Laboratory for Endocrine and Metabolic Diseases of the National Health Commission of the PR China, Shanghai Key Laboratory for Endocrine Tumor, Shanghai Digital Medicine Innovation Center, Ruijin Hospital, Shanghai Jiao Tong University School of Medicine, Shanghai 200025, China; Department of Endocrine and Metabolic Diseases, Shanghai Institute of Endocrine and Metabolic Diseases, Ruijin Hospital, Shanghai Jiao Tong University School of Medicine, Shanghai 200025, China; Shanghai National Clinical Research Center for Metabolic Diseases, Key Laboratory for Endocrine and Metabolic Diseases of the National Health Commission of the PR China, Shanghai Key Laboratory for Endocrine Tumor, Shanghai Digital Medicine Innovation Center, Ruijin Hospital, Shanghai Jiao Tong University School of Medicine, Shanghai 200025, China; Department of Endocrine and Metabolic Diseases, Shanghai Institute of Endocrine and Metabolic Diseases, Ruijin Hospital, Shanghai Jiao Tong University School of Medicine, Shanghai 200025, China; Shanghai National Clinical Research Center for Metabolic Diseases, Key Laboratory for Endocrine and Metabolic Diseases of the National Health Commission of the PR China, Shanghai Key Laboratory for Endocrine Tumor, Shanghai Digital Medicine Innovation Center, Ruijin Hospital, Shanghai Jiao Tong University School of Medicine, Shanghai 200025, China; MRC Integrative Epidemiology Unit, Bristol Medical School, University of Bristol, Oakfield House, Bristol BS8 2BN, UK; Department of Endocrine and Metabolic Diseases, Shanghai Institute of Endocrine and Metabolic Diseases, Ruijin Hospital, Shanghai Jiao Tong University School of Medicine, Shanghai 200025, China; Shanghai National Clinical Research Center for Metabolic Diseases, Key Laboratory for Endocrine and Metabolic Diseases of the National Health Commission of the PR China, Shanghai Key Laboratory for Endocrine Tumor, Shanghai Digital Medicine Innovation Center, Ruijin Hospital, Shanghai Jiao Tong University School of Medicine, Shanghai 200025, China; Department of Endocrine and Metabolic Diseases, Shanghai Institute of Endocrine and Metabolic Diseases, Ruijin Hospital, Shanghai Jiao Tong University School of Medicine, Shanghai 200025, China; Shanghai National Clinical Research Center for Metabolic Diseases, Key Laboratory for Endocrine and Metabolic Diseases of the National Health Commission of the PR China, Shanghai Key Laboratory for Endocrine Tumor, Shanghai Digital Medicine Innovation Center, Ruijin Hospital, Shanghai Jiao Tong University School of Medicine, Shanghai 200025, China; SJTU-Ruijin-UIH Institute for Medical Imaging Technology, Ruijin Hospital, Shanghai Jiao Tong University School of Medicine, Shanghai 200025, China; Department of Endocrine and Metabolic Diseases, Shanghai Institute of Endocrine and Metabolic Diseases, Ruijin Hospital, Shanghai Jiao Tong University School of Medicine, Shanghai 200025, China; Shanghai National Clinical Research Center for Metabolic Diseases, Key Laboratory for Endocrine and Metabolic Diseases of the National Health Commission of the PR China, Shanghai Key Laboratory for Endocrine Tumor, Shanghai Digital Medicine Innovation Center, Ruijin Hospital, Shanghai Jiao Tong University School of Medicine, Shanghai 200025, China; Department of Endocrine and Metabolic Diseases, Shanghai Institute of Endocrine and Metabolic Diseases, Ruijin Hospital, Shanghai Jiao Tong University School of Medicine, Shanghai 200025, China; Shanghai National Clinical Research Center for Metabolic Diseases, Key Laboratory for Endocrine and Metabolic Diseases of the National Health Commission of the PR China, Shanghai Key Laboratory for Endocrine Tumor, Shanghai Digital Medicine Innovation Center, Ruijin Hospital, Shanghai Jiao Tong University School of Medicine, Shanghai 200025, China; Department of Endocrine and Metabolic Diseases, Shanghai Institute of Endocrine and Metabolic Diseases, Ruijin Hospital, Shanghai Jiao Tong University School of Medicine, Shanghai 200025, China; Shanghai National Clinical Research Center for Metabolic Diseases, Key Laboratory for Endocrine and Metabolic Diseases of the National Health Commission of the PR China, Shanghai Key Laboratory for Endocrine Tumor, Shanghai Digital Medicine Innovation Center, Ruijin Hospital, Shanghai Jiao Tong University School of Medicine, Shanghai 200025, China; Department of Endocrine and Metabolic Diseases, Shanghai Institute of Endocrine and Metabolic Diseases, Ruijin Hospital, Shanghai Jiao Tong University School of Medicine, Shanghai 200025, China; Shanghai National Clinical Research Center for Metabolic Diseases, Key Laboratory for Endocrine and Metabolic Diseases of the National Health Commission of the PR China, Shanghai Key Laboratory for Endocrine Tumor, Shanghai Digital Medicine Innovation Center, Ruijin Hospital, Shanghai Jiao Tong University School of Medicine, Shanghai 200025, China; Department of Endocrine and Metabolic Diseases, Shanghai Institute of Endocrine and Metabolic Diseases, Ruijin Hospital, Shanghai Jiao Tong University School of Medicine, Shanghai 200025, China; Shanghai National Clinical Research Center for Metabolic Diseases, Key Laboratory for Endocrine and Metabolic Diseases of the National Health Commission of the PR China, Shanghai Key Laboratory for Endocrine Tumor, Shanghai Digital Medicine Innovation Center, Ruijin Hospital, Shanghai Jiao Tong University School of Medicine, Shanghai 200025, China; Department of Endocrine and Metabolic Diseases, Shanghai Institute of Endocrine and Metabolic Diseases, Ruijin Hospital, Shanghai Jiao Tong University School of Medicine, Shanghai 200025, China; Shanghai National Clinical Research Center for Metabolic Diseases, Key Laboratory for Endocrine and Metabolic Diseases of the National Health Commission of the PR China, Shanghai Key Laboratory for Endocrine Tumor, Shanghai Digital Medicine Innovation Center, Ruijin Hospital, Shanghai Jiao Tong University School of Medicine, Shanghai 200025, China; Department of Endocrine and Metabolic Diseases, Shanghai Institute of Endocrine and Metabolic Diseases, Ruijin Hospital, Shanghai Jiao Tong University School of Medicine, Shanghai 200025, China; Shanghai National Clinical Research Center for Metabolic Diseases, Key Laboratory for Endocrine and Metabolic Diseases of the National Health Commission of the PR China, Shanghai Key Laboratory for Endocrine Tumor, Shanghai Digital Medicine Innovation Center, Ruijin Hospital, Shanghai Jiao Tong University School of Medicine, Shanghai 200025, China; Department of Endocrine and Metabolic Diseases, Shanghai Institute of Endocrine and Metabolic Diseases, Ruijin Hospital, Shanghai Jiao Tong University School of Medicine, Shanghai 200025, China; Shanghai National Clinical Research Center for Metabolic Diseases, Key Laboratory for Endocrine and Metabolic Diseases of the National Health Commission of the PR China, Shanghai Key Laboratory for Endocrine Tumor, Shanghai Digital Medicine Innovation Center, Ruijin Hospital, Shanghai Jiao Tong University School of Medicine, Shanghai 200025, China; Department of Endocrine and Metabolic Diseases, Shanghai Institute of Endocrine and Metabolic Diseases, Ruijin Hospital, Shanghai Jiao Tong University School of Medicine, Shanghai 200025, China; Shanghai National Clinical Research Center for Metabolic Diseases, Key Laboratory for Endocrine and Metabolic Diseases of the National Health Commission of the PR China, Shanghai Key Laboratory for Endocrine Tumor, Shanghai Digital Medicine Innovation Center, Ruijin Hospital, Shanghai Jiao Tong University School of Medicine, Shanghai 200025, China; Department of Endocrine and Metabolic Diseases, Shanghai Institute of Endocrine and Metabolic Diseases, Ruijin Hospital, Shanghai Jiao Tong University School of Medicine, Shanghai 200025, China; Shanghai National Clinical Research Center for Metabolic Diseases, Key Laboratory for Endocrine and Metabolic Diseases of the National Health Commission of the PR China, Shanghai Key Laboratory for Endocrine Tumor, Shanghai Digital Medicine Innovation Center, Ruijin Hospital, Shanghai Jiao Tong University School of Medicine, Shanghai 200025, China; Department of Endocrine and Metabolic Diseases, Shanghai Institute of Endocrine and Metabolic Diseases, Ruijin Hospital, Shanghai Jiao Tong University School of Medicine, Shanghai 200025, China; Shanghai National Clinical Research Center for Metabolic Diseases, Key Laboratory for Endocrine and Metabolic Diseases of the National Health Commission of the PR China, Shanghai Key Laboratory for Endocrine Tumor, Shanghai Digital Medicine Innovation Center, Ruijin Hospital, Shanghai Jiao Tong University School of Medicine, Shanghai 200025, China; Department of Endocrine and Metabolic Diseases, Shanghai Institute of Endocrine and Metabolic Diseases, Ruijin Hospital, Shanghai Jiao Tong University School of Medicine, Shanghai 200025, China; Shanghai National Clinical Research Center for Metabolic Diseases, Key Laboratory for Endocrine and Metabolic Diseases of the National Health Commission of the PR China, Shanghai Key Laboratory for Endocrine Tumor, Shanghai Digital Medicine Innovation Center, Ruijin Hospital, Shanghai Jiao Tong University School of Medicine, Shanghai 200025, China; School of Public Health, Li Ka Shing Faculty of Medicine, The University of Hong Kong, Hong Kong Special Administration Region 999077, China; School of Public Health, Li Ka Shing Faculty of Medicine, The University of Hong Kong, Hong Kong Special Administration Region 999077, China; Department of Endocrine and Metabolic Diseases, Shanghai Institute of Endocrine and Metabolic Diseases, Ruijin Hospital, Shanghai Jiao Tong University School of Medicine, Shanghai 200025, China; Shanghai National Clinical Research Center for Metabolic Diseases, Key Laboratory for Endocrine and Metabolic Diseases of the National Health Commission of the PR China, Shanghai Key Laboratory for Endocrine Tumor, Shanghai Digital Medicine Innovation Center, Ruijin Hospital, Shanghai Jiao Tong University School of Medicine, Shanghai 200025, China; Department of Endocrine and Metabolic Diseases, Shanghai Institute of Endocrine and Metabolic Diseases, Ruijin Hospital, Shanghai Jiao Tong University School of Medicine, Shanghai 200025, China; Shanghai National Clinical Research Center for Metabolic Diseases, Key Laboratory for Endocrine and Metabolic Diseases of the National Health Commission of the PR China, Shanghai Key Laboratory for Endocrine Tumor, Shanghai Digital Medicine Innovation Center, Ruijin Hospital, Shanghai Jiao Tong University School of Medicine, Shanghai 200025, China; MRC Integrative Epidemiology Unit, Bristol Medical School, University of Bristol, Oakfield House, Bristol BS8 2BN, UK

**Keywords:** Mendelian randomization analysis, SGLT2 inhibition, chronological age, biological age, cognitive function, intelligence, brain imaging-derived phenotypes

## Abstract

**Introduction:**

An observational study suggested sodium-glucose cotransporter 2 (SGLT2) inhibitors might promote healthy aging. However, whether brain-related phenotypes mediate this association is still a question. We applied Mendelian randomization (MR) to investigate the effect of SGLT2 inhibition on chronological age, biological age, and cognition and explore the mediation effects of brain imaging-derived phenotypes (IDPs).

**Methods:**

We selected genetic variants associated with both expression levels of *SLC5A2* (Genotype-Tissue Expression and eQTLGen data; n = 129 to 31 684) and hemoglobin A1c (HbA1c) levels (UK Biobank; n = 344 182) and used them to proxy the effect of SGLT2 inhibition. Aging-related outcomes, including parental longevity (n = 389 166) and epigenetic clocks (n = 34 710), and cognitive phenotypes, including cognitive function (n = 300 486) and intelligence (n = 269 867) were derived from genome-wide association studies. Two-step MR was conducted to explore the associations between SGLT2 inhibition, IDPs, and aging outcomes and cognition.

**Results:**

SGLT2 inhibition was associated with longer father's attained age [years of life increase per SD (6.75 mmol/mol) reduction in HbA1c levels = 6.21, 95% confidence interval (CI) 1.27-11.15], better cognitive function (beta = .17, 95% CI 0.03-0.31), and higher intelligence (beta = .47, 95% CI 0.19-0.75). Two-step MR identified 2 IDPs as mediators linking SGLT2 inhibition with chronological age (total proportion of mediation = 22.6%), where 4 and 5 IDPs were mediators for SGLT2 inhibition on cognitive function and intelligence, respectively (total proportion of mediation = 61.6% and 68.6%, respectively).

**Conclusion:**

Our study supported that SGLT2 inhibition increases father's attained age, cognitive function, and intelligence, which was mediated through brain images of different brain regions. Future studies are needed to investigate whether a similar effect could be observed for users of SGLT2 inhibitors.

With trends in global aging, the elderly population has brought heavy medical and economic burdens (1). Healthy aging is currently one of the key priorities in global health research (2), given aging is associated with several noncommunicable diseases, including cardiovascular disease, cancers, and brain-related diseases (3).

Extending healthy longevity using drug intervention is the primary aim in drug development. Recent observational studies and clinical trials are investigating the causal effect of first-line antidiabetic drugs, such as metformin, on aging and aging-related diseases (4-6). For instance, recent Mendelian randomization (MR) studies have provided good evidence to support the effect of metformin targets on cardiovascular diseases, cancer, and dementia (7, 8). Other antidiabetic medications, such as sodium-glucose cotransporter 2 (SGLT2) inhibitors, also appear to have an effect independent of their glycemic properties and are currently being considered for drug reposition to promote healthy longevity (9). SGLT2 inhibitors are a novel class of antidiabetic drugs for type 2 diabetes mellitus treatment (10). The mechanism of this medication involves reducing glucose reuptake from the proximal tubules, thus promoting glucose excretion in the urine (11). In addition to their glucose-lowering effect, SGLT2 inhibitors were associated with better kidney outcomes and lower cardiovascular events and all-cause mortality in patients with or without type 2 diabetes mellitus (12-15). As such, the postulate that SGLT2 inhibitors might promote healthy aging has recently been proposed (16), including both chronological and biological age (17).

Of note, cognitive abilities, such as conceptual reasoning, memory, and speed of processing, are essential for older people's functional independence and quality of life but often level off with normal aging (18). Also, some studies have regarded cognitive dysfunction as a complication of diabetes, since people with diabetes are at higher risk of cognitive impairment (19). As such, we can hypothesize that SGLT2 inhibition may have an effect on cognitive function and thus promote healthy aging in the older population. At the same time, aging and high glucose concentrations bring inevitable changes to the brain (20, 21). It is foreseeable that the associations between SGLT2 inhibition and cognition may be mediated by changes in brain structures. Identifying specific regions mediating SGLT2 inhibition-dependent cognitive and intelligence changes may allow for the development of radiomic predictors of cognitive impairment in individuals with diabetes or even in healthy populations.

Although the use of randomized controlled trials to interrogate the effect of SGLT2i on longevity and mental health conditions can provide a more definitive answer, this often requires long-term follow-up. MR is an approach that uses germline genetic variants as instruments to estimate the causal effect of an exposure (eg, SGLT2 inhibition) on an outcome (eg, aging). Notably, earlier MR studies have identified the potential protective effect of SGLT2 inhibition on coronary artery disease (22). In this study, we investigated the casual effect of genetically proxied SGLT2 inhibition on chronological age, biological age, and cognition using 2-sample MR. To provide mechanistic insights, we used 2-step MR to understand whether the effect of SGLT2 inhibition influences aging and cognition by changes in brain structures.

## Methods

### Study Design

**Figure 1.**
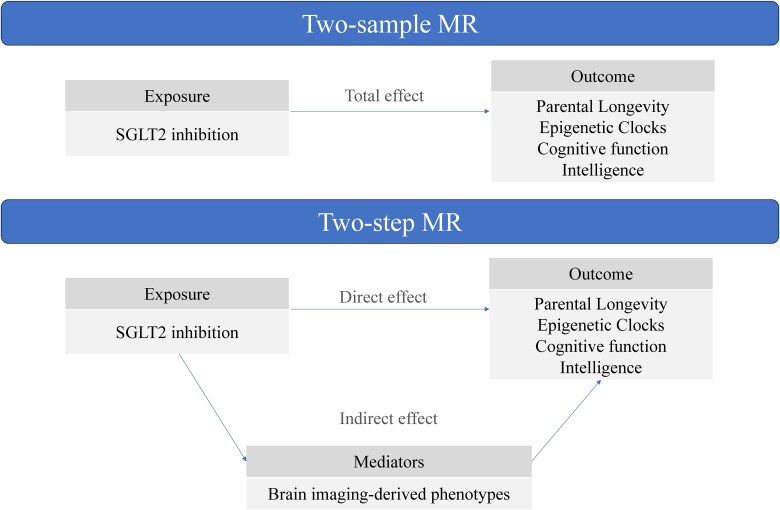
Study design. We first selected genetic variants for SGLT2 inhibition. Then parental longevity (father's attained age, mother's attained age, and combined parental attained age), epigenetic clocks (HannumAge, Intrinsic Epigenetic Age Acceleration, PhenoAge, and GrimAge), were chosen as study outcomes to reflect aging. Cognitive function and intelligence were selected as cognitive phenotypes. We conducted a 2-sample MR to investigate the casual role of SGLT2 inhibition on aging-related outcomes, cognitive function, and intelligence. A 2-step MR was applied to understand whether the effect of SGLT2 inhibition influences aging and cognition by brain IDPs. The IVW was applied as the primary method in the MR analysis. Abbreviations: IDP, brain imaging-derived phenotype; IVW, inverse variance weighted; MR, Mendelian randomization; SGLT2, sodium-glucose cotransporter 2.

The design of this study is presented in Fig. 1. First, we selected instruments for SGLT2 inhibition and estimated the casual effects of SGLT2 inhibition on aging-related outcomes, cognitive function, and intelligence using MR. Second, we selected brain imaging-derived phenotypes (IDPs) as mediators, and for each IDP, we chose corresponding genetic variants to act as proxies. Third, we conducted a 2-step MR to test the effects between SGLT2 inhibition, brain IDPs, and aging outcomes, cognitive function, and intelligence.

### Selection for Genetic Predictors of SGLT2 Inhibition

We applied a stringent instrument selection approach to identify genetic variants that best proxy SGLT2 inhibition (23), which updated our previous selection process (22). This process has 3 steps. First, genetic variants associated with expression levels of the target gene of SLGT2 (*SLC5A2*) at a regional-wide association threshold (*P* < .001) and hemoglobin A1c (HbA1c) levels (*P* < 5 × 10^−7^) within the *SLC5A2* region (±1Mb window) were filtered. Expression quantitative trait loci data were derived from Genotype-Tissue Expression (24) and the eQTLGen Consortium (25) (n = 129 to 31 684). The genetic association data of HbA1c were extracted from the UK Biobank. Second, we selected genetic variants that showed genetic colocalization evidence (colocalization probability > 0.7) between HbA1c and expression levels of *SLC5A2* as valid instruments, since these variants are more likely to be real causal variants, which were more likely to be assumed as valid instruments. Third, we clumped variants at a linkage disequilibrium r^2^ threshold of 0.15 to remove variants with strong correlations between each other. After these selection processes, we selected 2 genetic variants as valid instruments to proxy SGLT2 inhibition. The genetic associations of these variants were extracted from a genome-wide association studies (GWAS) of HbA1c (n = 344 182; Neale Lab UK Biobank GWAS release V2) (Table 1).

**Table 1.** The instrumental variables selected for SGLT2 inhibition

Phenotype	SNP	Effect_allele	Other_allele	Effect_allele_freq	Beta	Se	*P*	N	F-statistics
SGLT2	rs9930811	G	A	0.365	−.016	0.002	8.69E-12	344 182	/
SGLT2	rs35445454	T	C	0.344	−.013	0.002	1.24E-07	344 182	33.55

Abbreviations: SGLT2, sodium-glucose cotransporter 2; SNP, single nucleotide polymorphism.

The F-statistics of the instruments proxying SGLT2 inhibition and HbA1c have been calculated to estimate the instrument strength of the genetic predictors.

### Study Outcomes

We chose 2 sets of aging-related phenotypes and 2 cognitive phenotypes as outcomes for this study. First, parental longevity phenotypes, including father's attained age, mother's attained age, and combined parental attained age (n = 389 166), were chosen as outcomes to reflect chronological age. Studying longevity outcomes from first-degree relatives provides a more effective approach since the offspring with genetic data in a cohort share 50% of their DNA with their parents. Second, we selected 4 epigenetic clock phenotypes (n = 34 710) as another set of outcomes to reflect biological age, which is one of the most plausible candidates of phenotypic aging. Epigenetic clocks are biomarkers of aging developed using DNA methylation-level data at different Cytosine-phosphate-Guanine sites (26), which can predict chronological age with strong accuracy and reflect aging. The first generation of epigenetic aging clocks includes HannumAge (27) and Intrinsic HorvathAge (28), and the second generation includes PhenoAge (29) and GrimAge (30). All the genetic associations with these outcomes were obtained from a GWAS of chronological age (31) and epigenetic clocks (32).

Third, cognitive function was selected as a major outcome that reflects the state of brain function. The GWAS summary statistics of cognitive function were leveraged from Davies et al (33), including 300 486 individuals of European ancestry from a meta-analysis of 57 population-based cohorts, which were brought together by the Cohorts for Heart and Aging Research in Genomic Epidemiology, the Cognitive Genomics Consortium, and the UK Biobank. For the Cohorts for Heart and Aging Research in Genomic Epidemiology and Cognitive Genomics Consortium cohorts, cognitive function was constructed from a number of cognitive tasks. Due to the different measurements applied to all samples, a consistent method of extracting a general cognitive function component from cognitive test data was applied in these 2 cohorts. For participants of the UK Biobank, a fluid cognitive test was performed. In addition, intelligence was also considered a major study outcome of this study given its strong link with cognitive function. Genetic associations of intelligence were extracted from a genome-wide association meta-analysis of 14 cohorts (n = 269 867) (34). Different measures of intelligence were operationalized to index a common latent *g* factor underlying multiple dimensions of cognitive functioning.

### Selection of Mediators

With the increasingly emerging GWAS of brain IDPs (35) (defined as distinct measures of brain structure and function), the identification of brain IDPs using MR become possible. Given the association between aging-related outcomes and brain structure changes, we selected a total of 1366 structural magnetic resonance imaging (MRI) IDPs and diffusion MRI-related IDPs as candidate mediators to reflect brain structures (Supplementary Table S1) (36). A GWAS performed by Lloyd T. Elliott, including 8428 subjects from the UK Biobank, was used as the source of summary statistics for the selected IDPs (35). When considering IDPs as exposures in the 2-step MR, uncorrelated instruments (r^2^ < 0.001) for IDPs were selected at *P* < 1 × 10^−5^, since limited genome-wide association signals can be identified with the current sample size.

For all summary data used in our analyses, a detailed description of the participants enrolled in each study is listed in Supplementary Note 1 (36).

### Statistical Analyses

#### Main MR analysis of SGLT2 inhibition on aging-related outcomes

In the main analysis, a 2-sample MR approach was employed to estimate the causal effects of SGLT2 inhibition on aging-related outcomes. Owing to the fact that there were only 2 genetic predictors proxying SGLT2 inhibition, the causal effect on these outcomes was estimated with the random effect inverse variance weighted (IVW) method. To assess heterogeneity, Cochran's Q test was conducted utilizing IVW methods (*P* for Cochran's Q test < .05).

#### Two-step MR analysis

We applied a 2-step MR to validate whether the effect of SGLT2 inhibition on aging-related outcomes and cognitive phenotypes was through changes in brain-related phenotypes. In step 1, we estimated the effects of SGLT2 inhibition (as an exposure) on potential mediators, including 1336 IDPs (as outcomes). In step 2, SGLT2 inhibition-associated brain IDPs were considered potential mediators and were included as exposures in this analysis. The effects of each of the brain-related phenotypes (as an exposure) on aging-related outcomes and cognitive phenotypes (as outcomes) were estimated.

IVW, weighted median, simple mode, weighted mode, and MR-Egger regression were performed to estimate the causal relationships between these exposures and outcomes in each step, of which IVW was set as the primary analysis. The product method and the delta method were applied to calculate the indirect effect and the standard error of the indirect effect for the mediation MR, respectively (37). The mediation proportion was calculated as the indirect effect divided by the total effect.

#### Evaluation of MR assumptions

This study followed the Strengthening the Reporting of Observational Studies in Epidemiology Using Mendelian Randomization guidelines (38, 39). MR has 3 core assumptions: (1) the genetic variants must be robustly associated with the exposure, (2) the genetic variants should not be associated with any common causes (genetic confounders) between the instrument and the outcome, and (3) the genetic variants must be associated with the outcome only via the exposure. By measuring the strength of genetic variants using the F-statistic, we test the potential violation of the first MR assumption. A common threshold (F-statistic > 10) was used for the confirmation of robust instruments (40). In the sensitivity analysis, the intercept test for MR-Egger was applied to detect the potential pleiotropic effects, indicating the existence of pleiotropy at *P* for intercept <.05. For the assessment of heterogeneity, the Cochran's Q test was conducted utilizing MR-Egger regression and IVW methods (*P* for heterogeneity <.05).

To visualize the results for the primary outcome, when presenting MR estimates, we multiply by 2.2869 for the father's attained age to interpret the genetic associations given offspring only share 50% of DNA with parents, then multiply by 10 to meet with a longstanding actuarial rule of thumb (41). In addition, we present our results by inversing the direction of the effects of SGLT2 inhibition on outcomes to make it easy to understand, which is in accordance with the glycemic-lowering effect of SGLT2 inhibition.

All analyses were implemented using the “TwoSampleMR”? package and “ieugwasr”? package in R software version 4.2.2. Results of MR were reported as beta with SE and were considered statistically significant at *P* < .05.

## Results

### Instruments Strength of SGLT2 Inhibition

To quantify the statistical power of the genetic variants, we estimated the strength of the genetic predictors of each exposure using F-statistics. The strength of the instruments was above 10, which suggested that weak instrument bias is unlikely to affect this MR study (Table 1).

### The Effect of SGLT2 Inhibition on Aging and Cognitive Outcomes

**Figure 2.**
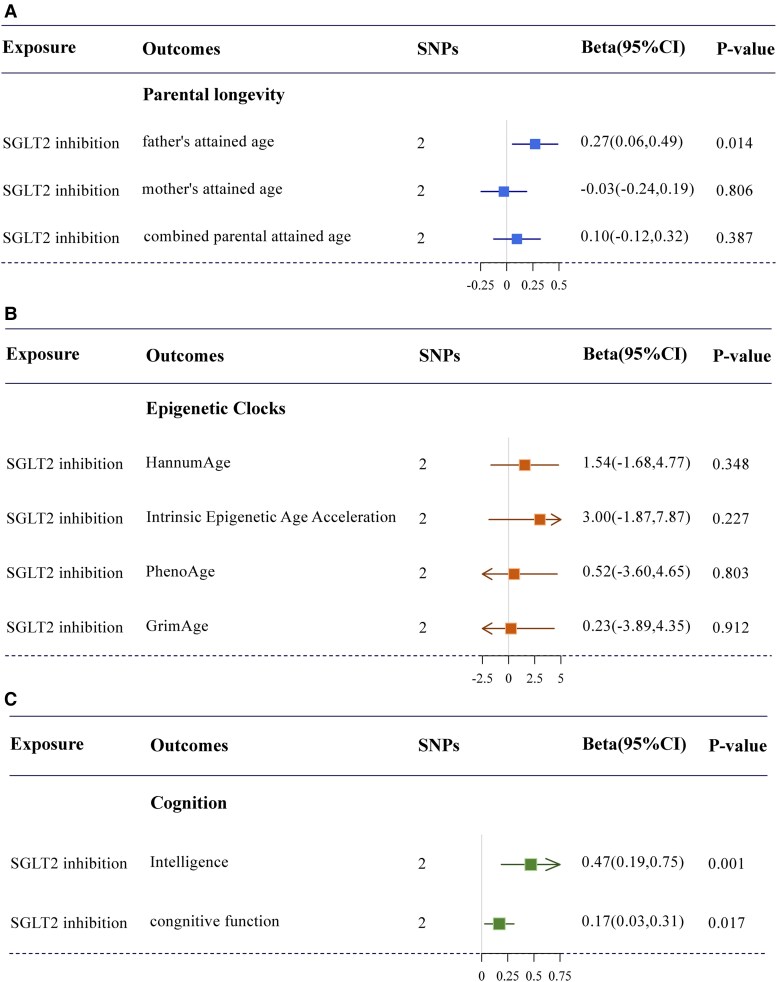
Casual effect of SGLT2 inhibition on parental longevity, epigenetic clocks and cognition. (A) Causal effects of SGLT2 inhibition on parental longevity. (B) Causal effects of SGLT2 inhibition on epigenetic clocks. (C) Causal effects of SGLT2 inhibition on cognitive function. MR estimates are expressed as the effect of per SD (6.75 mmol/mol) reduction in HbA1c via SGLT2 inhibition on parental longevity (father's attained age, mother's attained age, and combined parental attained age), epigenetic clocks (HannumAge, Intrinsic Epigenetic Age Acceleration, PhenoAge, and GrimAge), and cognition (cognitive function and intelligence) with the IVW method. Abbreviations: CI, confidence interval; IVW, inverse-variance weighted; MR, Mendelian randomization; SGLT2, sodium-glucose cotransporter 2; SNP, single nucleotide polymorphism.

For chronological age, SGLT2 inhibition showed an effect on longer father's attained age [years of life increase, 6.21, 95% confidence interval (CI) 1.27-11.15, *P* = .01] (Fig. 2A). But we observed little evidence to support the effect of SGLT2 inhibition on mother's attained age and combined parental attained age (Supplementary Table S2) (36). For biological age, there was little evidence indicating the causality of SGLT2 inhibition on HannumAge, Intrinsic HorvathAge, PhenoAge, and GrimAge (Fig. 2B and Supplementary Table S2) (36) For all MR results, little evidence was observed to support the existence of heterogeneity (*P-*value of Cochran's Q statistic >.05).

We found a protective effect of SGLT2 inhibition on cognitive function (beta = .17, 95% CI 0.03-0.31, *P* = .02) and intelligence (beta = .47, 95% CI 0.19-0.75, *P* = .001) (Fig. 2C and Supplementary Table S2) (36).

### The Effect of SGLT2 Inhibition on 1366 Brain IDPs

SGLT2 inhibition showed associations with 98 brain IDPs, such as isotropic volume fraction in the right corticospinal tract (beta = 1.69, 95% CI 0.13-3.25, *P* = .03) and diffusion tensor mode of the left superior longitudinal fasciculus (beta = 2.38, 95% CI 0.43-4.33, *P* = .02). The direction of effect for these results were consistent across all sensitivity analyses (Supplementary Table S3) (36).

### The Effect of Brain IDPs on Aging-related Outcomes

**Figure 3.**
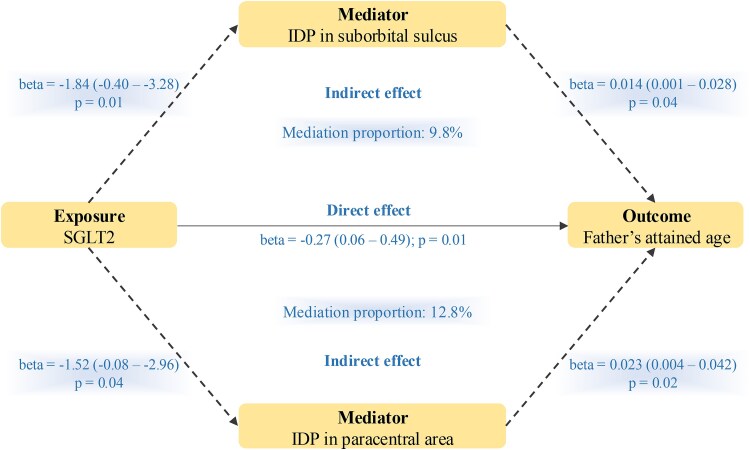
Mediation analysis of the effect of SGLT2 inhibition on father's attained age via IDPs in the 2-step MR framework. Two-step MR was used to evaluate the mediating role of IDPs in the associations of SGLT2 inhibition with father's attained age. The product method and delta method were applied to calculate the indirect effect and the standard error of the effect, respectively. Mediation proportion is calculated as indirect effect divided by direct (total) effect. The IVW method was applied as the primary method. Abbreviations: IDP, imaging-derived phenotype; IDP in suborbital sulcus, a2009s rh S suborbital area; IDP in paracentral area, DKTatlas lh paracentral thickness; IVW, inverse variance weighted; MR, Mendelian randomization; SGLT2, sodium-glucose cotransporter 2.

IDPs need to be associated with both SGLT2 inhibition and father's attained age, and they must show directionally consistent results in the 2-step MR analysis (eg, SGLT2 inhibition was negatively associated with 1 IDP; this IDP was positively associated with father's attained age, where SGLT2 inhibition was negatively associated with father's attained age). Under this criterion, 2 IDPs were identified from the aforementioned 98 IDPs. SGLT2 inhibition was associated with changes in the right supraorbital sulcus area (beta = 1.84, 95% CI 0.40-3.28, *P* = .01) and thickness of the paracentral area in the left hemisphere (beta = 1.52, 95% CI 0.08-2.96, *P* = .04), both of which led to an increase in father's attained age (beta = .014, 95% CI 0.001-0.028, *P* = .04; beta = .023, 95% CI 0.004-0.042, *P* = .02). (Fig. 3 and Supplementary Table S4) (36). An indirect effect via these 2 IDPs accounted for 9.8% and 12.8% of the total effect between SGLT2 inhibition and father's attained age (Supplementary Table S5) (36). Little evidence was found to support the existence of heterogeneity and pleiotropy (Supplementary Table S4) (36). In addition, of the 98 IDPs associated with SGLT2 inhibition, 7 and 6 of them showed an association with mother's attained age and combined parental attained age, respectively. Due to the inconclusive association between SGLT2 inhibition on mother's attained age and combined parental attained age, we were not able to conduct a directional consistency test and mediation analysis (Supplementary Table S4) (36).

### The Effect of IDPs on Cognitive Function and Intelligence

Of the 98 SGLT2 inhibition-associated IDPs, 4 and 6 IDPs were robustly associated with cognitive function and intelligence, respectively. For example, SGLT2 inhibition showed an effect on increased thickness superior and transverse occipital sulcus (beta = 1.77, 95% CI 0.10-3.44, *P* = .04), which was associated with better cognitive function in the meantime (beta = .013, 95% CI 0.002-0.024, *P* = .02). (Supplementary Table S6) (36). There was evidence for an indirect effect of SGLT2 inhibition on cognitive function through this trait (beta = .023, 95% CI −0.006 to 0.052), accounting for 13.5% of the total effect. (Supplementary Table 7) (36). Another example was that SGLT2 inhibition was associated with a thicker superior part of the precentral sulcus (beta = 2.27, 95% CI 0.83-3.71, *P* = .002), which led to the increase in intelligence (beta = .028, 95% CI 0.004-0.052, *P* = .02). In a sensitivity analysis, we found evidence to suggest the existence of heterogeneity in the casual estimates of 2 IDPs on cognitive function and 2 IDPs on intelligence. Meanwhile, MR-Egger regression implied the evidence of directional pleiotropy in the association between 1 IDP and intelligence. (Supplementary Table S6) (36). Finally, after excluding the MR result containing pleiotropy, 4 and 5 IDPs remained, respectively. Given the correlation between cognitive function and intelligence, we observed whether there was an overlap between the IDPs. We found 3 IDPs overlapped, which were in the cerebral peduncle, superior and transverse occipital sulcus, and superior part of the precentral sulcus. As such, the reliability of our results is further increased.

## Discussion

In this study, we estimated the effect of SGLT2 inhibition on chronological age, biological age, cognitive function, and intelligence using MR. Our results suggested that genetically predicted SGLT2 inhibition may increase father's attained age, where 2 IDPs mediated the effect of SGLT2 inhibition on father’s attained age. Our 2-step MR also suggested that SGLT2 inhibition had a beneficial effect on the maintenance of cognitive function and intelligence. We further identified 4 and 5 brain IDPs mediated the effect of SGLT2 inhibition on cognitive function and intelligence, respectively.

One key finding of our study is that SGLT2 inhibition increased father's attained age, which implies that SGLT2 inhibition may increase lifespans for males. This aligns with findings from a previous animal study, which indicated that canagliflozin, 1 type of SGLT2 inhibitor, could extend life expectancy, and the effect was only seen in male mice (42). Meanwhile, a meta-analysis suggested that treatment with SGLT2 inhibitors could result in a reduction in all-cause death (43), which aligns with our findings.

The beneficial effect of SGLT2 inhibition on lifespan can be mediated by many factors. Of note, SGLT2 inhibitors reduced the risk of chronic diseases such as heart failure and chronic kidney disease, which accounts for a considerable proportion of mortality (44, 45). However, it is not directly clear whether brain-related phenotypes showed a mediation role for SGLT2 inhibitors in aging. We have identified 2 IDPs located in the supraorbital sulcus and paracentral area of the brain that may mediate the effects between SGLT2 inhibition and father's attained age. These new results provide an attractive pathway linking SGLT2 inhibition with aging via brain imaging traits. Few additional studies have explored the relationship between specific brain regions and longer human longevity. Therefore, we still need to interpret our findings with caution. Further studies are needed to replicate these associations and discover the causal mechanisms.

Our study also found a potential effect of SGLT2 inhibition on maintaining cognitive function. A previous randomized controlled trial has been performed in elderly diabetic individuals. No obviously reduced cognitive performance was found in patients who received SGLT2 inhibitors for 12 months (46). However, only 50 patients were recruited, which made it hard to draw a reliable conclusion. Meanwhile, animal models showed that the canagliflozin's ameliorating effects have been evaluated in rats with scopolamine-induced memory dysfunction (47). The mechanisms underlying the neuroprotective effect of SGLT2 inhibitors have also been investigated before. An experimental study found that empagliflozin could attenuate cerebral oxidative stress and DNA oxidative damage in obese and diabetic mice (48). Meanwhile, it could increase mice's cerebral brain-derived neurotrophic factor levels, which are believed to have a crucial role in synaptic plasticity and memory but are always less in diabetic patients (49, 50). Therefore, SGLT2 inhibitors may increase brain-derived neurotrophic factor levels in humans. All of these studies support our findings of the effect of SGLT2 inhibition on cognitive function and intelligence. The therapeutic potential of SGLT2 inhibitors for maintaining cognitive performance in an aging population is worth further investigation.

Our 2-step MR further showed that several brain image phenotypes mediated the effect of SGLT2 inhibition on cognitive function, which provided medical image evidence to support that SGLT2 inhibition may influence brain structure and further influence cognition. For instance, 1 IDP, located in the cerebral peduncle, was influenced by

SGLT2 inhibition and was further associated with both cognitive function and intelligence. A previous diffusion tensor imaging study reported the relationship between cerebellum peduncles and cognitive functions such as sustained attention and working memory, which was consistent with our findings (51, 52). On the other hand, we found an IDP in the superior and transverse occipital sulcus as a mediator. The relationship between the transverse occipital sulcus and visual cognition such as scene perception has already been shown, and this region was found to be activated in visual processing (53, 54). Further mechanistic studies of the effects of SGLT2 inhibition on cognitive function and intelligence are required to confirm the functions of these relevant brain regions.

Our study has several limitations. First, aging outcomes are complex and challenging to measure using genetic tools directly. Therefore, we do not recommend interpreting our results as a direct causal effect of SGLT2 inhibition on delaying aging. Instead, the effect of SGLT2 inhibition may influence some intermediate phenotypes, for example, factors that influence MRI image phenotypes in certain brain regions and indirectly increase longevity. Further functional analyses are needed to better understand how the underlying mechanisms of SGLT2 inhibition influence brain image traits. Second, the estimates may be biased due to partial sample overlap between the exposure and outcome data, given both GWAS data contained participants from the UK Biobank. Since the genetic variants we used to proxy SGLT2 inhibition have good instrument strength (F-statistic = 33.55), such bias is minimized in our main analyses (55). Third, notably, we have used parental longevity as a surrogate for the participant's survival condition, which means the casual estimates generated by our instruments could be diluted (56). The accuracy of using parental longevity to proxy individuals' lifespan can be disputed, as it can be influenced by various cardiometabolic, social, and lifestyle factors. Fourth, our analysis did not find evidence of biologically aging-delaying effects related to SGLT2 inhibition. However, in contrast to our result, mechanisms underlying this potential effect, such as modulation of nutrient-sensing pathways (57-60) and reduction in the interleukin-6 (61) inflammatory mediator, both of which are associated with many aging-related diseases, have already been suggested in previous studies. Therefore, the effect of SGLT2 inhibition on delaying aging may still exist despite our negative results. Fifth, using a genetic variant in *SLC5A2* to proxy the pharmacological SGLT2 inhibition, a recent study reported the cardioprotective effects of SGLT2 inhibition (62). However, the effects may not be attributed to lower plasma glucose since only a minimal mediation proportion was found. As our instruments were selected based on the HbA1c-lowering effect of SGLT2 inhibition, we should view our results with caution. Finally, the populations of the exposure and outcome data are all of European descent. While bias due to population stratification has been minimized, the generalizability of our findings to other ethnic groups is restricted.

## Conclusions

In conclusion, our study found evidence to support the protective effect of SGLT2 inhibition on increasing lifespans for men and supporting the maintenance of cognition. The life-expending and cognition-protecting effect of SGLT2 inhibition through changes in brain structures provides an attractive causal pathway for SGLT2 inhibition, which is worth further investigation in future functional studies and clinical trials.

## Data Availability

Only summary statistics have been used in this study. Details of the data source are listed in Supplementary Table S8 (36).

## Permission Statement

Only summary statistics have been used in this study. All participants provided informed consent at enrollment.
